# Crystal structure of 3-bromo-9-ethyl-9*H*-carbazole

**DOI:** 10.1107/S2056989015023907

**Published:** 2015-12-19

**Authors:** Mykola Bezuglyi, Gintare Grybauskaite, Gintautas Bagdziunas, Juozas Vidas Grazulevicius

**Affiliations:** a Department of Chemistry, National Taras Shevchenko University, 62a Volodymirska st., Kyiv, Ukraine; b Department of Polymer Chemistry and Technology, Kaunas University of Technology, Radvilenu Road 19, LT-50254, Kaunas, Lithuania

**Keywords:** crystal structure, carbazole, C—H⋯π inter­actions

## Abstract

In the title compound, C_14_H_12_BrN, the tricyclic ring system is essentially planar (r.m.s. deviation 0.026 Å). The carbon atoms of the ethyl group deviate from the mean plane by 0.148 (9) (CH_2_) and 1.59 (1) Å (CH_3_). In the crystal, H⋯π contacts [2.698–2.898 Å] shorter than the van der Waals contact distance of 3.70 Å are observed. A scalable to gram quantities selective synthesis of mono-bromine-substituted carbazole derivatives was developed.

## Related literature   

*N*-substituted carbazole derivatives are important for anti-cancer research (Caulfield *et al.*, 2002 ▸) and as materials for opto-electronic devices (Niu *et al.*, 2011 ▸; Miyazaki *et al.*, 2014 ▸; Grigalevicius *et al.*, 2002 ▸). The crystal structure of 1,3,6,8-tetra­bromo-9-ethyl-9*H*-carbazole was reported by Bezuglyi *et al.* (2015 ▸).
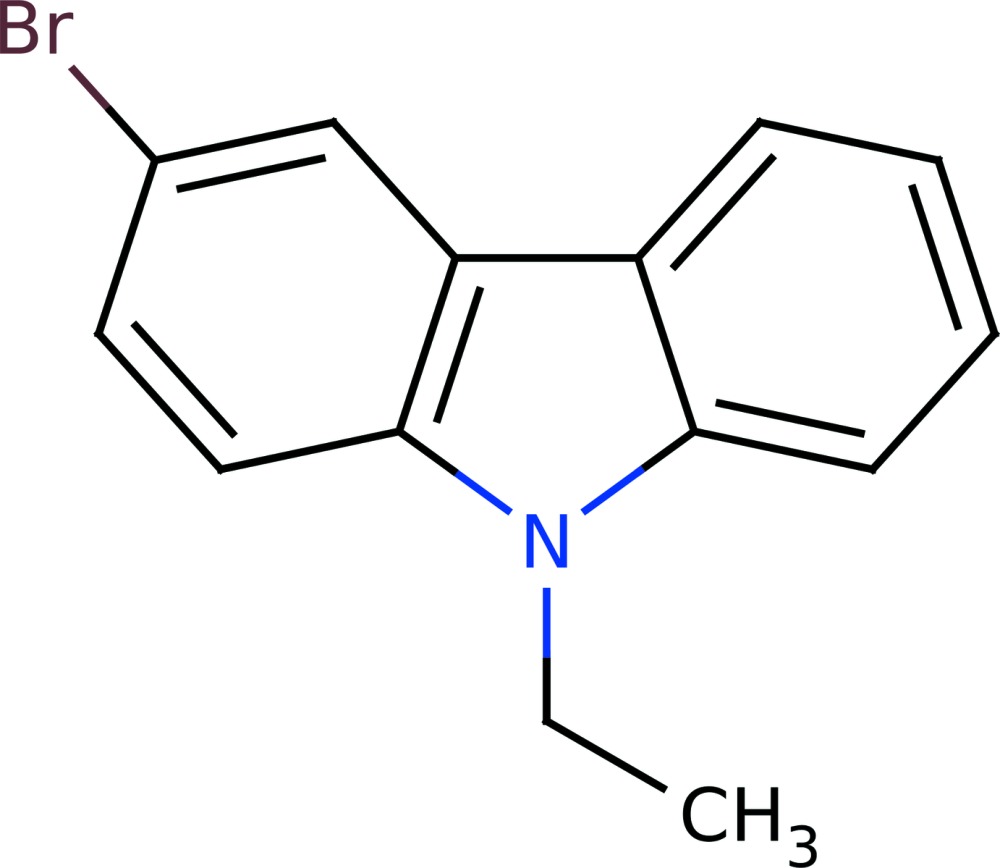


## Experimental   

### Crystal data  

C_14_H_12_BrN
*M* *_r_* = 274.16
Orthorhombic, 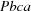

*a* = 15.263 (16) Å
*b* = 7.745 (8) Å
*c* = 20.41 (2) Å
*V* = 2413 (5) Å^3^
*Z* = 8
Mo *K*α radiation
μ = 3.39 mm^−1^
*T* = 293 K
0.40 × 0.09 × 0.08 mm

### Data collection  

Rigaku XtaLAB mini diffractometer
Absorption correction: multi-scan (*REQAB*; Rigaku, 1998 ▸) *T*_min_ = 0.450, *T*_max_ = 0.763
8316 measured reflections
2721 independent reflections
1383 reflections with *F* ^2^ > 2.0σ(*F* ^2^)
*R*_int_ = 0.056

### Refinement  

*R*[*F* ^2^ > 2σ(*F* ^2^)] = 0.078
*wR*(*F* ^2^) = 0.236
*S* = 1.05
2721 reflections
145 parameters
H-atom parameters constrained
Δρ_max_ = 1.37 e Å^−3^
Δρ_min_ = −0.46 e Å^−3^

### 

Data collection: *CrystalClear-SM Expert* (Rigaku, 2011 ▸); cell refinement: *CrystalClear-SM Expert*; data reduction: *CrystalClear-SM Expert*; program(s) used to solve structure: *SHELXS97* (Sheldrick, 2008 ▸); program(s) used to refine structure: *SHELXL97* (Sheldrick, 2008 ▸); molecular graphics: *ORTEP-3 for Windows* (Farrugia, 2012 ▸); software used to prepare material for publication: *CrystalStructure* (Rigaku, 2010 ▸).

## Supplementary Material

Crystal structure: contains datablock(s) General, I. DOI: 10.1107/S2056989015023907/nk2233sup1.cif

Structure factors: contains datablock(s) I. DOI: 10.1107/S2056989015023907/nk2233Isup2.hkl

Supporting information file. DOI: 10.1107/S2056989015023907/nk2233Isup3.cml

**Fig. 1.**
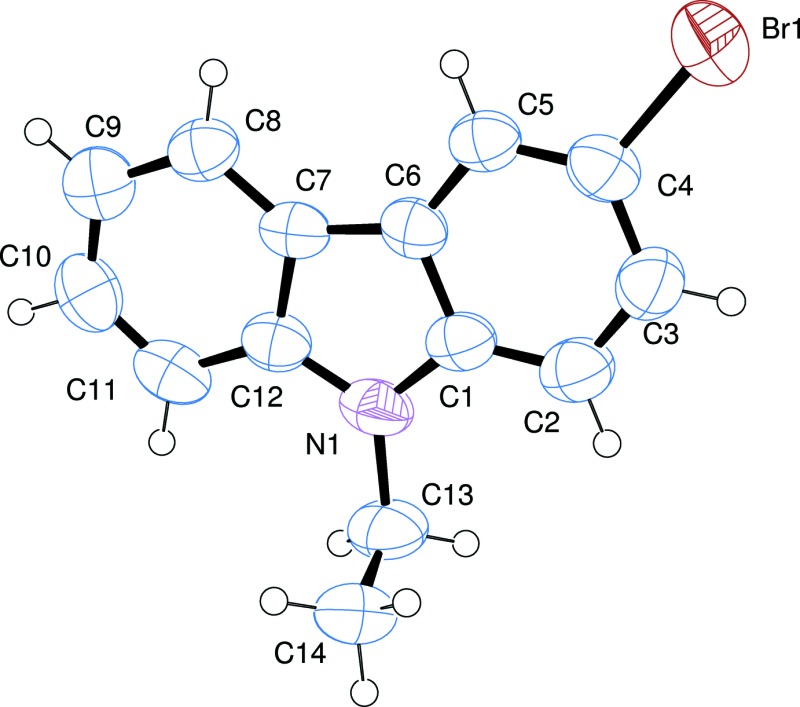
The molecular structure of the title molecule with displacement ellipsoids drawn at the 50% probability level.

. DOI: 10.1107/S2056989015023907/nk2233fig1.tif
The mol­ecular structure of the title mol­ecule with displacement ellipsoids drawn at the 50% probability level.

CCDC reference: 1442215

Additional supporting information:  crystallographic information; 3D view; checkCIF report

## Figures and Tables

**Table 1:** Hydrogen-bond geometry (Å, °) *Cg*1 are the centroids of the N1/C1/C6/C7/C12 and C1–C6 rings, respectively.

*D*—H⋯*A*	*D*—H	H⋯*A*	*D*⋯*A*	*D*—H⋯*A*
C8—H8⋯*Cg*1^i^	0.93	2.81	3.637 (7)	149
C11—H11⋯*Cg*2^ii^	0.93	3.01	3.922 (8)	167

Symmetry code: (i) 
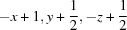
; (ii) 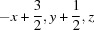.

## supplementary crystallographic information

### S1. Synthesis and crystallization

9-ethyl-carbazole (1.00 g, 5.12 mmol) was added to a solution of *N*-bromo­succinimide (0.911 g, 5.12 mmol) in 10 ml of DMF. The reaction mixture was refluxed at room temperature for 24 h. When the reaction was completed (monitored *via* TLC) the solution was poured into a large amount of water with ice and extracted with ethyl acetate. The organic layer was dried over anhydrous sodium sulfate followed by solvent evaporation in rotary evaporator. The product was crystallized from methanol to afford a white needle-like crystals. Yield: 0.88 g (62%), melting point 58–60°C. ^1^H NMR (700 MHz, CDCl_3_) δ 8.10 (d, *J* = 7.7 Hz, 1H), 7.63 (d, *J* = 2.5 Hz, 1H), 7.49 (ddd, *J* = 8.2, 7.1, 1.1 Hz, 1H), 7.42 (d, *J* = 8.2 Hz, 1H), 7.35 (d, *J* = 8.7 Hz, 1H), 7.25 – 7.22 (m, 1H), 7.16 (dd, *J* = 8.8, 2.5 Hz, 1H), 4.37 (q, *J* = 7.3 Hz, 2H), 1.45 (t, *J* = 7.3 Hz, 4H).

### S2. Refinement

All hydrogen atoms were placed in geometrically idealized positions and constrained to ride on their parent atoms, with C—H = 0.930 Å for aromatic C—H, with 0.969 Å for methyl­ene C—H, 0.957 Å for methyl distances and *U*_iso_(H) = 1.2 *U*_eq_.

### Crystal data

**Table:**

C_14_H_12_BrN	*F*(000) = 1104.00
*M**_r_* = 274.16	*D*_x_ = 1.509 Mg m^−^^3^
Orthorhombic, *P**b**c**a*	Mo *K*α radiation, λ = 0.71075 Å
Hall symbol: -P 2ac 2ab	Cell parameters from 3894 reflections
*a* = 15.263 (16) Å	θ = 3.1–27.5°
*b* = 7.745 (8) Å	µ = 3.39 mm^−^^1^
*c* = 20.41 (2) Å	*T* = 273 K
*V* = 2413 (5) Å^3^	Chip, colorless
*Z* = 8	0.40 × 0.09 × 0.08 mm

### Data collection

**Table:**

Rigaku XtaLAB mini diffractometer	1383 reflections with *F*^2^ > 2.0σ(*F*^2^)
Detector resolution: 13.653 pixels mm^-1^	*R*_int_ = 0.056
ω scans	θ_max_ = 27.5°
Absorption correction: multi-scan (*REQAB*; Rigaku, 1998)	*h* = −18→18
*T*_min_ = 0.450, *T*_max_ = 0.763	*k* = −10→7
8316 measured reflections	*l* = −26→21
2721 independent reflections	

### Refinement

**Table:**

Refinement on *F*^2^	Secondary atom site location: difference Fourier map
*R*[*F*^2^ > 2σ(*F*^2^)] = 0.078	Hydrogen site location: inferred from neighbouring sites
*wR*(*F*^2^) = 0.236	H-atom parameters constrained
*S* = 1.05	*w* = 1/[σ^2^(*F*_o_^2^) + (0.0926*P*)^2^ + 4.1576*P*] where *P* = (*F*_o_^2^ + 2*F*_c_^2^)/3
2721 reflections	(Δ/σ)_max_ < 0.001
145 parameters	Δρ_max_ = 1.37 e Å^−^^3^
0 restraints	Δρ_min_ = −0.46 e Å^−^^3^
Primary atom site location: structure-invariant direct methods	

### Special details

**Table:**

Geometry. ENTER SPECIAL DETAILS OF THE MOLECULAR GEOMETRY
Refinement. Refinement was performed using all reflections. The weighted *R*-factor (*wR*) and goodness of fit (*S*) are based on *F*^2^. *R*-factor (gt) are based on *F*. The threshold expression of *F*^2^ > 2.0 σ(*F*^2^) is used only for calculating *R*-factor (gt).

### Fractional atomic coordinates and isotropic or equivalent isotropic displacement parameters (Å^2^)

**Table:**

	*x*	*y*	*z*	*U*_iso_*/*U*_eq_	
Br1	0.28798 (6)	0.61687 (12)	0.45898 (4)	0.0841 (5)	
N1	0.6116 (4)	0.2371 (7)	0.3684 (3)	0.0635 (15)	
C1	0.5364 (5)	0.3073 (8)	0.3969 (4)	0.0570 (17)	
C2	0.4935 (6)	0.2633 (10)	0.4542 (4)	0.0680 (19)	
C3	0.4189 (6)	0.3557 (10)	0.4719 (4)	0.070 (2)	
C4	0.3891 (5)	0.4915 (9)	0.4327 (4)	0.0629 (18)	
C5	0.4317 (5)	0.5360 (9)	0.3759 (4)	0.0559 (16)	
C6	0.5065 (5)	0.4456 (8)	0.3573 (4)	0.0527 (16)	
C7	0.5643 (4)	0.4553 (8)	0.3016 (4)	0.0519 (16)	
C8	0.5686 (5)	0.5583 (9)	0.2455 (4)	0.0608 (18)	
C9	0.6333 (5)	0.5278 (10)	0.1987 (4)	0.071 (2)	
C10	0.6935 (5)	0.3978 (11)	0.2092 (4)	0.072 (3)	
C11	0.6939 (5)	0.2950 (10)	0.2632 (5)	0.067 (2)	
C12	0.6283 (5)	0.3243 (9)	0.3100 (4)	0.0577 (17)	
C13	0.6556 (6)	0.0790 (9)	0.3911 (4)	0.074 (3)	
C14	0.6145 (6)	−0.0826 (9)	0.3648 (5)	0.083 (3)	
H3	0.4107	0.6258	0.3500	0.0671*	
H8	0.7363	0.3795	0.1775	0.0866*	
H9	0.6358	0.5943	0.1608	0.0853*	
H10	0.5139	0.1737	0.4803	0.0816*	
H13	0.3888	0.3269	0.5100	0.0840*	
H14	0.5283	0.6470	0.2394	0.0730*	
H15	0.7358	0.2091	0.2688	0.0804*	
H17A	0.6458	−0.1813	0.3809	0.0995*	
H17B	0.6167	−0.0812	0.3178	0.0995*	
H17C	0.5545	−0.0887	0.3789	0.0995*	
H18A	0.6540	0.0757	0.4386	0.0894*	
H18B	0.7166	0.0827	0.3778	0.0894*	

### Atomic displacement parameters (Å^2^)

**Table:**

	*U*^11^	*U*^22^	*U*^33^	*U*^12^	*U*^13^	*U*^23^
Br1	0.0778 (7)	0.0894 (7)	0.0852 (7)	0.0084 (5)	0.0147 (5)	−0.0107 (5)
N1	0.067 (4)	0.053 (3)	0.071 (4)	0.017 (3)	−0.015 (3)	−0.007 (3)
C1	0.071 (5)	0.043 (4)	0.056 (4)	−0.003 (4)	−0.017 (4)	−0.005 (3)
C2	0.080 (5)	0.059 (4)	0.065 (5)	0.004 (4)	−0.012 (4)	0.004 (4)
C3	0.091 (6)	0.063 (5)	0.056 (5)	−0.006 (5)	0.001 (4)	0.001 (4)
C4	0.062 (5)	0.053 (4)	0.074 (5)	0.000 (4)	0.002 (4)	−0.008 (4)
C5	0.061 (4)	0.045 (4)	0.061 (4)	−0.003 (4)	−0.010 (4)	−0.001 (3)
C6	0.055 (4)	0.045 (4)	0.058 (4)	−0.003 (3)	−0.007 (4)	−0.009 (3)
C7	0.049 (4)	0.043 (4)	0.063 (4)	−0.000 (3)	−0.017 (3)	−0.007 (3)
C8	0.062 (4)	0.056 (4)	0.065 (5)	−0.001 (4)	−0.011 (4)	0.003 (4)
C9	0.069 (5)	0.069 (5)	0.075 (5)	−0.011 (5)	−0.001 (4)	−0.005 (4)
C10	0.064 (5)	0.083 (6)	0.070 (5)	−0.006 (5)	−0.002 (4)	−0.021 (5)
C11	0.054 (4)	0.060 (5)	0.087 (6)	0.007 (4)	−0.014 (4)	−0.019 (4)
C12	0.054 (4)	0.050 (4)	0.068 (5)	0.001 (4)	−0.007 (4)	−0.004 (4)
C13	0.080 (5)	0.064 (5)	0.080 (5)	0.020 (4)	−0.023 (5)	0.007 (4)
C14	0.104 (7)	0.051 (4)	0.094 (6)	0.010 (5)	0.001 (5)	0.003 (4)

### Geometric parameters (Å, º)

**Table:**

Br1—C4	1.901 (7)	C10—C11	1.358 (12)
N1—C1	1.396 (9)	C11—C12	1.403 (11)
N1—C12	1.393 (10)	C13—C14	1.499 (11)
N1—C13	1.471 (10)	C2—H10	0.930
C1—C2	1.382 (10)	C3—H13	0.930
C1—C6	1.418 (9)	C5—H3	0.930
C2—C3	1.392 (12)	C8—H14	0.930
C3—C4	1.397 (11)	C9—H9	0.930
C4—C5	1.373 (10)	C10—H8	0.930
C5—C6	1.391 (9)	C11—H15	0.930
C6—C7	1.442 (10)	C13—H18A	0.970
C7—C8	1.397 (10)	C13—H18B	0.970
C7—C12	1.419 (9)	C14—H17A	0.960
C8—C9	1.394 (11)	C14—H17B	0.960
C9—C10	1.381 (11)	C14—H17C	0.960
			
N1···C5	3.594 (9)	C10···H3^vi^	2.9041
N1···C8	3.594 (10)	C10···H15^ix^	2.9077
C1···C4	2.761 (10)	C11···H3^vi^	3.1000
C1···C14	3.311 (11)	C11···H14^vi^	3.5811
C2···C5	2.811 (10)	C11···H15^ix^	3.3834
C2···C13	3.133 (12)	C11···H17A^ix^	3.4343
C3···C6	2.781 (11)	C11···H17B^ix^	3.2429
C5···C8	3.388 (10)	C11···H18B^ix^	3.5072
C7···C10	2.764 (11)	C12···H14^vi^	2.9363
C8···C11	2.819 (10)	C12···H18B^ix^	3.3937
C9···C12	2.767 (11)	C13···H17A^ix^	3.5602
C11···C13	3.156 (12)	C14···H13^vii^	3.1805
C12···C14	3.351 (11)	C14···H14^x^	3.5604
C2···C3^i^	3.574 (11)	C14···H14^vi^	3.5260
C2···C4^i^	3.486 (11)	C14···H15^xi^	3.4162
C3···C2^i^	3.574 (11)	H3···C9^ii^	3.3372
C3···C3^i^	3.527 (12)	H3···C10^ii^	2.9041
C4···C2^i^	3.486 (11)	H3···C11^ii^	3.1000
Br1···H3	2.9084	H3···H8^ii^	3.0356
Br1···H13	2.9144	H3···H8^iii^	3.3234
N1···H10	2.7717	H3···H15^ii^	3.3615
N1···H15	2.7889	H3···H17C^viii^	3.1708
N1···H17A	3.2924	H8···Br1^vi^	3.4695
N1···H17B	2.6741	H8···Br1^xii^	3.4304
N1···H17C	2.6783	H8···C4^xii^	3.3551
C1···H3	3.2683	H8···C5^xii^	3.3993
C1···H13	3.2280	H8···C9^xi^	3.4012
C1···H17C	3.1012	H8···H3^vi^	3.0356
C1···H18A	2.6759	H8···H3^xii^	3.3234
C1···H18B	3.2769	H8···H9^xi^	2.9669
C2···H17C	3.2655	H8···H15^ix^	3.1884
C2···H18A	2.8668	H9···Br1^xii^	3.3766
C3···H3	3.2516	H9···C1^ii^	3.3199
C4···H10	3.2612	H9···C2^ii^	3.3337
C5···H13	3.2457	H9···C3^ii^	3.4827
C5···H14	3.2684	H9···C6^ii^	3.5009
C6···H10	3.2790	H9···H8^ix^	2.9669
C6···H14	2.8883	H9···H15^ix^	3.0795
C7···H3	2.8662	H9···H17C^ii^	3.3331
C7···H9	3.2568	H10···C4^i^	3.4733
C7···H15	3.3072	H10···H10^vii^	2.8396
C8···H3	3.2607	H10···H17C^vii^	3.1274
C8···H8	3.2241	H13···Br1^xiii^	3.3182
C9···H15	3.2540	H13···C1^i^	3.5975
C10···H14	3.2341	H13···C14^vii^	3.1805
C11···H9	3.2440	H13···H17A^vii^	2.5518
C11···H17B	3.3353	H13···H17C^vii^	3.0494
C11···H18B	2.8803	H13···H18A^vii^	3.3550
C12···H8	3.1954	H13···H18B^v^	3.5574
C12···H14	3.2644	H14···N1^ii^	3.1441
C12···H17B	3.1500	H14···C1^ii^	3.2025
C12···H18A	3.2783	H14···C6^ii^	3.0863
C12···H18B	2.6882	H14···C7^ii^	2.8977
C13···H10	2.9205	H14···C8^ii^	3.5256
C13···H15	2.9579	H14···C11^ii^	3.5811
C14···H10	3.4427	H14···C12^ii^	2.9363
C14···H15	3.5181	H14···C14^viii^	3.5604
H3···H14	2.8901	H14···C14^ii^	3.5260
H8···H9	2.2887	H14···H17B^viii^	2.9697
H8···H15	2.2820	H14···H17B^ii^	3.0636
H9···H14	2.3299	H14···H17C^viii^	3.5302
H10···H13	2.3284	H14···H17C^ii^	3.2796
H10···H17C	2.9668	H15···C8^xi^	3.2416
H10···H18A	2.4234	H15···C9^xi^	2.8304
H15···H17B	3.0595	H15···C10^xi^	2.9077
H15···H18B	2.4483	H15···C11^xi^	3.3834
H17A···H18A	2.3159	H15···C14^ix^	3.4162
H17A···H18B	2.3126	H15···H3^vi^	3.3615
H17B···H18A	2.8071	H15···H8^xi^	3.1884
H17B···H18B	2.3301	H15···H9^xi^	3.0795
H17C···H18A	2.3268	H15···H17A^ix^	3.0376
H17C···H18B	2.8068	H15···H17B^ix^	2.9508
Br1···H8^ii^	3.4695	H17A···C3^vii^	3.4391
Br1···H8^iii^	3.4304	H17A···C7^x^	3.4783
Br1···H9^iii^	3.3766	H17A···C11^xi^	3.4343
Br1···H13^iv^	3.3182	H17A···C13^xi^	3.5602
Br1···H18A^i^	3.2895	H17A···H13^vii^	2.5518
Br1···H18A^v^	3.2825	H17A···H15^xi^	3.0376
N1···H14^vi^	3.1441	H17A···H18B^xi^	2.7853
C1···H9^vi^	3.3199	H17B···C8^x^	3.2433
C1···H13^i^	3.5975	H17B···C8^vi^	3.2915
C1···H14^vi^	3.2025	H17B···C11^xi^	3.2429
C2···H9^vi^	3.3337	H17B···H14^x^	2.9697
C3···H9^vi^	3.4827	H17B···H14^vi^	3.0636
C3···H17A^vii^	3.4391	H17B···H15^xi^	2.9508
C4···H8^iii^	3.3551	H17C···C5^x^	3.4596
C4···H10^i^	3.4733	H17C···C8^vi^	3.3569
C5···H8^iii^	3.3993	H17C···C9^vi^	3.3968
C5···H17C^viii^	3.4596	H17C···H3^x^	3.1708
C6···H9^vi^	3.5009	H17C···H9^vi^	3.3331
C6···H14^vi^	3.0863	H17C···H10^vii^	3.1274
C7···H14^vi^	2.8977	H17C···H13^vii^	3.0494
C7···H17A^viii^	3.4783	H17C···H14^x^	3.5302
C8···H14^vi^	3.5256	H17C···H14^vi^	3.2796
C8···H15^ix^	3.2416	H18A···Br1^i^	3.2895
C8···H17B^viii^	3.2433	H18A···Br1^xiv^	3.2825
C8···H17B^ii^	3.2915	H18A···H13^vii^	3.3550
C8···H17C^ii^	3.3569	H18B···C11^xi^	3.5072
C9···H3^vi^	3.3372	H18B···C12^xi^	3.3937
C9···H8^ix^	3.4012	H18B···H13^xiv^	3.5574
C9···H15^ix^	2.8304	H18B···H17A^ix^	2.7853
C9···H17C^ii^	3.3968		
			
C1—N1—C12	108.6 (6)	C1—C2—H10	120.636
C1—N1—C13	124.6 (6)	C3—C2—H10	120.638
C12—N1—C13	126.1 (6)	C2—C3—H13	119.841
N1—C1—C2	130.3 (7)	C4—C3—H13	119.848
N1—C1—C6	108.7 (6)	C4—C5—H3	120.246
C2—C1—C6	121.0 (7)	C6—C5—H3	120.252
C1—C2—C3	118.7 (7)	C7—C8—H14	120.069
C2—C3—C4	120.3 (7)	C9—C8—H14	120.061
Br1—C4—C3	119.2 (6)	C8—C9—H9	120.379
Br1—C4—C5	119.7 (6)	C10—C9—H9	120.368
C3—C4—C5	121.2 (7)	C9—C10—H8	118.092
C4—C5—C6	119.5 (6)	C11—C10—H8	118.084
C1—C6—C5	119.3 (6)	C10—C11—H15	121.478
C1—C6—C7	107.0 (6)	C12—C11—H15	121.484
C5—C6—C7	133.7 (6)	N1—C13—H18A	108.980
C6—C7—C8	134.8 (6)	N1—C13—H18B	108.983
C6—C7—C12	106.8 (6)	C14—C13—H18A	108.979
C8—C7—C12	118.4 (6)	C14—C13—H18B	108.984
C7—C8—C9	119.9 (7)	H18A—C13—H18B	107.770
C8—C9—C10	119.3 (8)	C13—C14—H17A	109.478
C9—C10—C11	123.8 (8)	C13—C14—H17B	109.463
C10—C11—C12	117.0 (7)	C13—C14—H17C	109.470
N1—C12—C7	108.9 (6)	H17A—C14—H17B	109.470
N1—C12—C11	129.5 (7)	H17A—C14—H17C	109.473
C7—C12—C11	121.6 (7)	H17B—C14—H17C	109.472
N1—C13—C14	113.0 (7)		
			
C1—N1—C12—C7	1.4 (7)	Br1—C4—C5—C6	179.0 (4)
C1—N1—C12—C11	−176.9 (6)	C3—C4—C5—C6	−0.8 (10)
C12—N1—C1—C2	179.1 (6)	C4—C5—C6—C1	1.0 (9)
C12—N1—C1—C6	−2.1 (7)	C4—C5—C6—C7	178.2 (6)
C1—N1—C13—C14	83.0 (8)	C1—C6—C7—C8	178.2 (6)
C13—N1—C1—C2	8.6 (11)	C1—C6—C7—C12	−1.1 (7)
C13—N1—C1—C6	−172.6 (6)	C5—C6—C7—C8	0.7 (13)
C12—N1—C13—C14	−85.8 (8)	C5—C6—C7—C12	−178.6 (7)
C13—N1—C12—C7	171.7 (6)	C6—C7—C8—C9	−177.5 (6)
C13—N1—C12—C11	−6.5 (11)	C6—C7—C12—N1	−0.1 (7)
N1—C1—C2—C3	179.7 (6)	C6—C7—C12—C11	178.3 (5)
N1—C1—C6—C5	179.9 (5)	C8—C7—C12—N1	−179.6 (6)
N1—C1—C6—C7	2.0 (7)	C8—C7—C12—C11	−1.2 (9)
C2—C1—C6—C5	−1.2 (10)	C12—C7—C8—C9	1.7 (9)
C2—C1—C6—C7	−179.1 (6)	C7—C8—C9—C10	−1.3 (10)
C6—C1—C2—C3	1.1 (10)	C8—C9—C10—C11	0.1 (12)
C1—C2—C3—C4	−0.8 (11)	C9—C10—C11—C12	0.4 (12)
C2—C3—C4—Br1	−179.2 (6)	C10—C11—C12—N1	178.1 (7)
C2—C3—C4—C5	0.6 (11)	C10—C11—C12—C7	0.1 (10)

Symmetry codes: (i) −*x*+1, −*y*+1, −*z*+1; (ii) −*x*+1, *y*+1/2, −*z*+1/2; (iii) *x*−1/2, *y*, −*z*+1/2; (iv) −*x*+1/2, *y*+1/2, *z*; (v) *x*−1/2, −*y*+1/2, −*z*+1; (vi) −*x*+1, *y*−1/2, −*z*+1/2; (vii) −*x*+1, −*y*, −*z*+1; (viii) *x*, *y*+1, *z*; (ix) −*x*+3/2, *y*+1/2, *z*; (x) *x*, *y*−1, *z*; (xi) −*x*+3/2, *y*−1/2, *z*; (xii) *x*+1/2, *y*, −*z*+1/2; (xiii) −*x*+1/2, *y*−1/2, *z*; (xiv) *x*+1/2, −*y*+1/2, −*z*+1.

### Hydrogen-bond geometry (Å, º)

Cg1 are the centroids of the N1/C1/C6/C7/C12 and C1–C6 rings, respectively.

**Table:**

*D*—H···*A*	*D*—H	H···*A*	*D*···*A*	*D*—H···*A*
C8—H8···*Cg*1^ii^	0.93	2.81	3.637 (7)	149
C11—H11···*Cg*2^ix^	0.93	3.01	3.922 (8)	167

Symmetry codes: (ii) −*x*+1, *y*+1/2, −*z*+1/2; (ix) −*x*+3/2, *y*+1/2, *z*.
