# Supplementary figures and images for: Crystal structure of 3-bromo-9-ethyl-9H-carbazole
Source: Acta Crystallogr E Crystallogr Commun. 2015 Dec 19;71(Pt 12):o1067–8. doi: 10.1107/S2056989015023907 (PMC4719982; doi:10.1107/S2056989015023907)

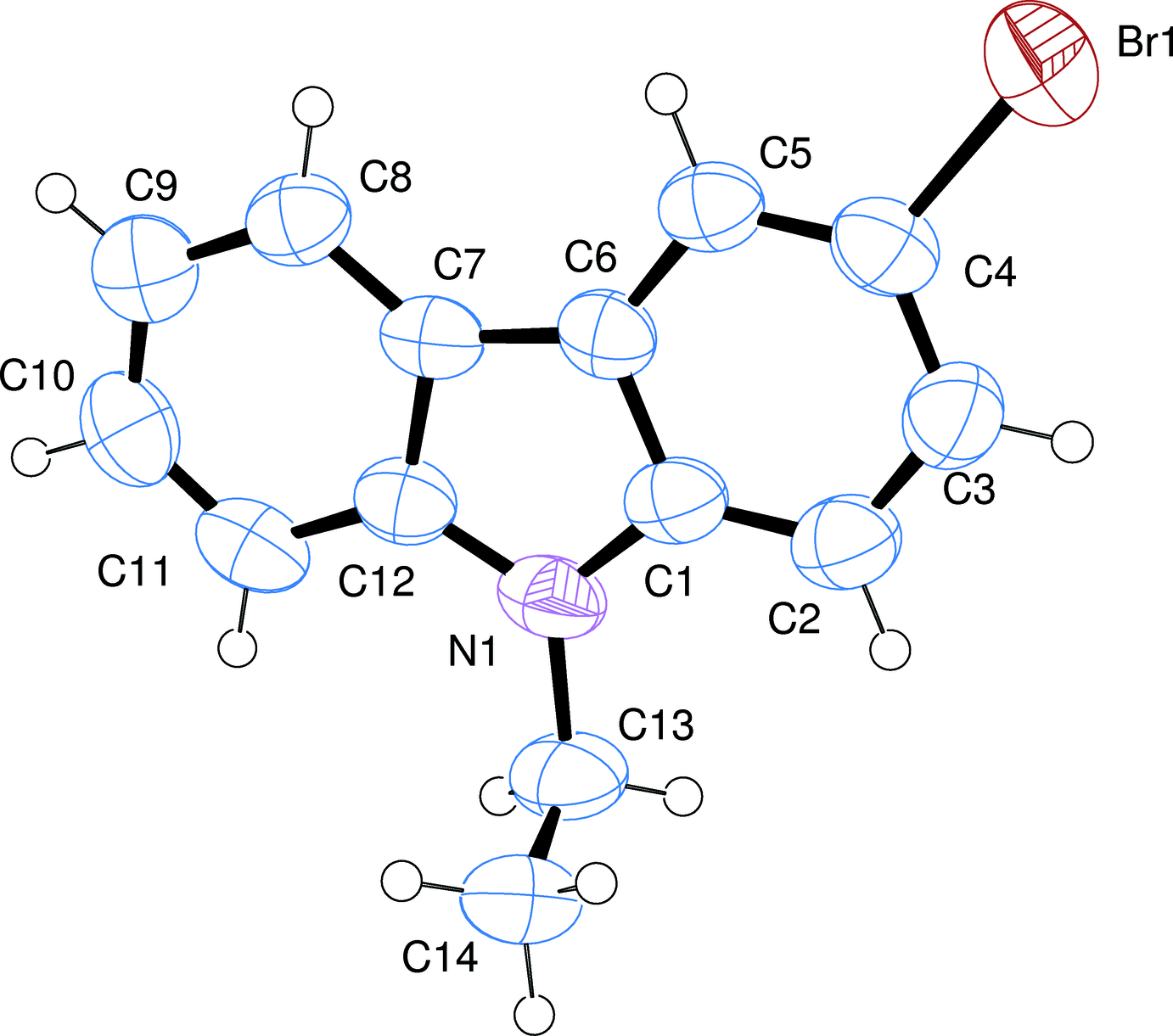

Supplement: Supplementary file 4 [file e-71-o1067-fig1.tif]
